# Paraesophageal Hernia in a Newborn Mimicking Esophageal Atresia

**DOI:** 10.7759/cureus.28655

**Published:** 2022-08-31

**Authors:** Zoi Lamprinou, Dimosthenis Chrysikos, George Tsakotos, Vasileios Protogerou, Theodore Troupis

**Affiliations:** 1 Anatomy, National and Kapodistrian University of Athens, Athens, GRC

**Keywords:** esophageal atresia, microgastria, neonate, congenital hiatal hernia, paraesophageal hernia

## Abstract

A defect of the esophageal hiatus can lead to herniation of the stomach or other abdominal organs into the thoracic cavity, a condition called hiatal hernia. They constitute a rare clinical entity during infancy and childhood and their symptoms can be non-specific or subtle, making the diagnosis difficult even for experienced clinicians. In all cases, surgical treatment of the defect is necessary because of life-threatening complications.

We present a rare case of a newborn with congenital paraesophageal hernia (CPEH) and microgastria, who was initially referred to our center with the diagnosis of esophageal atresia due to the inability to pass an orogastric tube beyond 15 cm from the gum margin. A contrast study revealed the CPEH. The patient underwent emergent surgery and has had no signs of recurrence until now.

Although the diagnosis can be very tricky and mimic other conditions, a high level of suspicion should exist especially in patients with persistent symptoms of gastroesophageal reflux or recurrent respiratory infections. In neonates, signs and symptoms can be indicative of esophageal obstruction which should be ruled out with an upper gastrointestinal (GI) study.

## Introduction

Hiatal hernia is one of the several known defects of the diaphragm and is well known to occur in adults. There are also a few cases of hiatal hernias described in the literature featuring younger patients. These cases present with non-specific symptoms such as vomiting, recurrent respiratory system infections, or failure to thrive but sometimes can have an acute clinical presentation with respiratory distress or gastric volvulus [1]. Congenital paraesophageal hernia (CPEH), a subtype of hiatal hernia in the newborn, is a very rare entity and the symptoms in some cases are suggestive of esophageal atresia or esophageal web. In these patients, upper gastrointestinal (GI) contrast studies can confirm the diagnosis.

Hiatal hernias occur when the stomach protrudes into the thoracic cavity through a widened gap, usually due to a developmental defect of the diaphragm. There are four types of esophageal hiatal hernias [1,2]: type I widely referred to as sliding hernia occurs when only the gastric cardia protrudes through the esophageal hiatus, types II-IV known as paraesophageal hernias (PEH), are less common. Type II occurs when the gastric fundus herniates through the hiatus but the gastroesophageal junction (GEJ) remains fixed in position. Type III is a combination of the previous two types in which the GEJ has an abnormal intrathoracic position and the gastric fundus protrudes into the thoracic cavity. Type IV is the paraesophageal hernia (PEH) that involves herniation of other abdominal organs such as the colon, small intestine, omentum, or spleen.

## Case presentation

**Figure 1:**
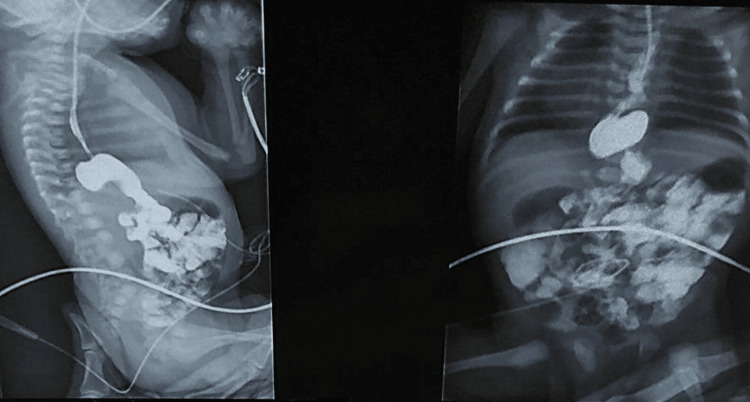
Upper GI study confirming the presence of paraesophageal hernia and microgastria GI: Gastrointestinal

A full-term male neonate (37 weeks and two days gestation) with the prenatal diagnosis of polyhydramnios was delivered by elective cesarean section with no evidence of respiratory distress. During the first feeding attempt, the baby had a choking episode with perioral cyanosis, and due to inability to insert the feeding tube to the stomach, the patient was instantly referred to our center with the prominent diagnosis of esophageal atresia. The investigation including chest X-ray and contrast study (Figure 1) revealed herniation of the whole stomach into the thoracic cavity and microgastria- type III PEH.

A laparotomy was decided and the patient was operated on the fourth day of life. The stomach was reduced in the abdomen and the hiatal defect was closed. A fundoplication could not be performed owing to microgastria. Thus, a gastrostomy tube was inserted to act as a gastropexy. The neonate began oral feeding the third postoperative day without incidents. Six weeks later, the gastrostomy tube was removed and since then the infant has normal growth with rare episodes of emesis.

## Discussion

Paraesophageal hernias in children, either congenital or acquired, are uncommon. The embryologic basis of CPEH is inadequately understood but there are several hypotheses [3]. During fetal development, the pneumoenteric recesses develop on either side of the midline of the mediastinum but the persistence of the right recess predisposes PEH development. Others have postulated that CPEH is due to laxity of the ligamentous attachments of the stomach and a deficient diaphragmatic hiatus. Alternatively, aberrant development of the lumbar component of the diaphragm which originates from mesodermal cells around the aorta can be considered as possible etiologies [1,4].

Patients with CPEH may surprisingly be asymptomatic or present with vague GI symptoms such as emesis and postprandial discomfort, recurrent respiratory infections, anemia, and failure to thrive. An assiduous medical history can also raise suspicion, especially in the asymptomatic child whose mother is worried about strange noises in the chest on swallowing, accompanied by chest symptoms or vomiting [5].

Yousef et al. [2] found that type II CPEH was more likely to present with gastroesophageal reflux symptoms even though respiratory symptoms were most commonly seen in types III and IV. The PEH in the neonatal period can be a tricky diagnosis, usually presenting with respiratory distress or symptoms indicative of high intestinal obstruction or esophageal atresia. Difficulty in passing a feeding tube, as described for our patient, which is usually arrested 14 cm to 15 cm from the gum margin, is a rare presentation of CPEH and can be differentially diagnosed from esophageal atresia where the level of resistance during insertion is higher [6].

Potentially life-threatening complications include gastric volvulus, bleeding, incarceration, obstruction, and gangrene or perforation. In a case series from Turkey [7], three out of five infants presented with intrathoracic gastric volvulus, a complication with significant morbidity and mortality.

Given the rare incidence and the vague nature of the symptoms, the diagnosis is often not initially suspected with the exception of the acute clinical presentation of the above-mentioned complications. The imaging evaluation begins with an anteroposterior and lateral chest X-ray (CXR). Karpelowsky et al. [4] confirmed the presence of an identifiable cystic mass in the posterior mediastinum in all 59 patients of their series who were diagnosed with PEH. Other suggestive findings are esophageal dilation and the abnormal position of the nasogastric tube in the thorax [1]. However, a normal CXR does not necessarily preclude the presence of PEH as there is the possibility of intermittent reduction.

Upper gastrointestinal contrast studies are the cornerstone of imaging as they can both confirm the diagnosis and also demonstrate complications. The pathognomonic findings are an 'upside down' stomach located intrathoracic while the GEJ usually remains intraabdominal [8]. The role of the CT scan remains unclear but this modality can help establish the diagnosis in doubtful cases. Other advantages are the assessment of the herniated contents and the exclusion of other pulmonary or mediastinal lesions [1].

Antenatal diagnosis of CPEH is described in some cases in the literature [1,9]. A fetal ultrasound can demonstrate a cystic mass in the posterior mediastinum between the heart and the thoracic spine, which can raise suspicion of the abnormality. A subsequent MRI scan of the fetus can confirm the diagnosis.

The diagnosis of CPEH is considered as an indication for surgical repair, regardless of the presence or absence of symptoms. A delay in surgery can lead to either symptom progression or complications that require emergency surgery with increased mortality [2,8]. A controversial issue concerning surgical management is the choice between the open and laparoscopic approaches. Although laparoscopic repair is well established in adults it is not commonly practiced in infants and children. Both Namgoon et al. in Korea and Petrosyan et al. in the USA, retrospectively compared the outcomes of the two methods in pediatric patients, pointing to no difference in the complications or length of stay. The mean operation time was longer for the laparoscopic method but the patients had a shorter time to oral intake [10,11]. Minimally invasive repair with the use of robotic surgery has been proposed as a feasible and safe alternative [12] but it has not yet gained popularity among pediatric surgeons.

Regardless of the approach, there are four cornerstones during the surgical repair of CPEH: 1) reduction of the hernia contents, 2) resection of the sac, 3) closure of the hiatal defect, and 4) an antireflux procedure. An antireflux procedure helps to both treat reflux disease that coexists in many cases and decreases the risk of recurrence [1, 7]. In our case, an antireflux procedure could not be performed due to microgastria.

## Conclusions

Paraesophageal hernia in children is a relatively rare entity and can occur at any age. It may be asymptomatic and be encountered incidentally or with vague symptoms such as recurrent chest infections or vomiting. In neonates, the situation can present with difficulty in inserting an orogastric feeding tube and can imitate esophageal atresia as in our case. Physicians caring for these patients should be aware of such a presentation as it has the potential for severe complications. Elective surgical treatment is mandatory shortly after the diagnosis due to the considerable morbidity and should include both hernia repair and an antireflux procedure.
